# Myocarditis Secondary to COVID-19 mRNA Vaccine: A Case Report

**DOI:** 10.7759/cureus.22345

**Published:** 2022-02-17

**Authors:** Lina M Mohammed, Vikramjeet Dhillon, Juyong Peter Bong, Jyothi Patri

**Affiliations:** 1 Family Medicine Residency Program, Adventist Health System-Central Valley Network, Hanford, USA

**Keywords:** post-vaccination, myocarditis, covid-19 disease, pericarditis, covid-19 mrna vaccine

## Abstract

Vaccine-induced myocarditis has been acknowledged in the past as a rare complication after vaccine administration including influenza and smallpox. Over the past year, there has been an increased number of myopericarditis cases reported by The Center for Disease Control and Prevention (CDC) following the administration of the BNT162b2 and mRNA-1273 vaccines. Most of these cases were among healthy young male adolescents. We report a case of myocarditis in a young male adolescent who presented with chest pain 2 weeks following the first dose of the mRNA COVID-19 vaccine. In the context of the COVID-19 pandemic, we believe it’s crucial for healthcare providers to recognize and consider myocarditis as a differential diagnosis in young otherwise healthy individuals who present with chest pain and cardiac symptoms.

## Introduction

Myocarditis is an inflammation of the myocardium that is mostly caused by viral etiology, primarily enteroviruses and adenoviruses such as (Coxsackie A, Coxsackie B, and echoviruses) [1]. In 2019, the global burden of the disease was found to be 6.1 and 4.4 per 100,000 in men and women, respectively [2]. It has been reported in the past following vaccination including influenza and smallpox [3]. Since the Food and Drug Administration (FDA) approved BNT162b2 and mRNA-1273 for emergency use authorization (EUA), there have been several cases of myocarditis following the administration of the COVID-19 mRNA vaccine [4]. Even though vaccine-related myocarditis is relatively rare [3], we believe it’s important for clinicians to recognize and consider it as a differential when a patient presents with a chest patient following the administration of the first or second dose of COVID-19 mRNA vaccine.

## Case presentation

**Figure 1:**
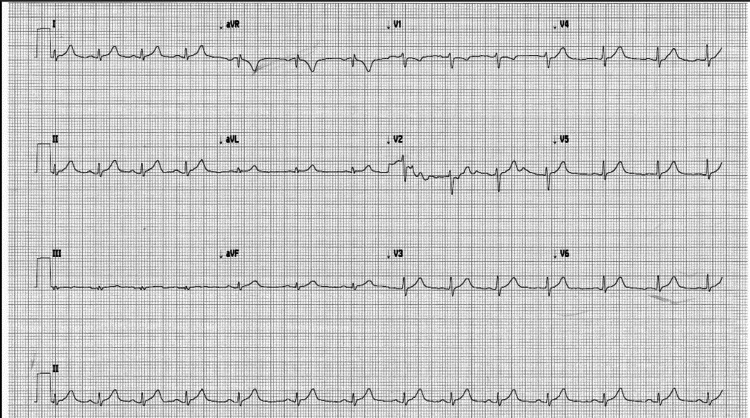
Electrocardiogram showing moderate ST depression in V1 and V2.

A previously healthy 19-year-old male presented to the Emergency Department for generalized chest pain of 2 days duration. He reported intermittent sharp diffuse pain, 7/10 in intensity, it started when he was asleep. The pain was worse when he lies flat associated with sweats. The patient denied any cough, fever, or shortness of breath, any recent illness, or knowing any sick person having COVID-19. He reported receiving the first dose of COVID-19 vaccine 2 weeks prior to his presentation. There was a previous history of COVID-19 infection one year ago (December 2020). On physical exam, the patient had a fever of 38.5°C, blood pressure of 123/71 mmHg, a pulse of 86 bpm, a respiratory rate of 18, and oxygen saturation of 98% on room air. The rest of the physical exam was normal (including a cardiac exam). An electrocardiogram (ECG) showed normal sinus rhythm with moderate ST depression in V1 and V2 (Figure 1).

**Figure 2:**
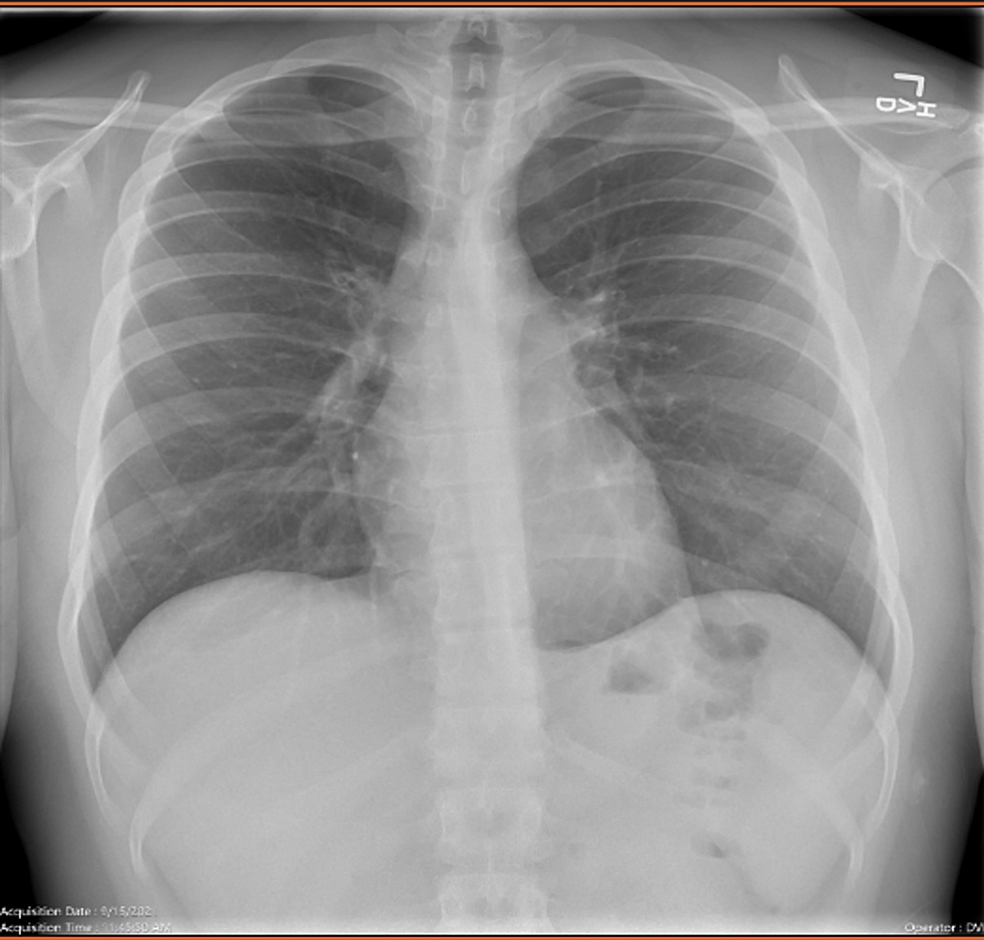
Chest X-ray showing normal cardio mediastinal silhouette. The pulmonary vasculature is unremarkable. ​No focal airspace opacity, pneumothorax, or pleural effusion is seen​.

Initial laboratory evaluation showed an elevated troponin I of 4.5 ng/mL (normal level <0.03 ng/mL), had an elevated erythrocyte sedimentation rate (ESR) of 36 mm/h (normal <15 mm/h), C-reactive protein (CRP) of 5.2 ng/mL (normal 0-0.5 ng/mL), and an elevated creatinine kinase (CK), total of 482 U/L (normal 22-198 U/L). severe acute respiratory syndrome coronavirus 2 (SARS-CoV-2) nasopharyngeal by PCR was performed and it was negative. Chest X-ray was performed which showed normal pulmonary vasculature, and cardiopulmonary silhouette was within the normal limits in size (Figure 2).

**Figure 3:**
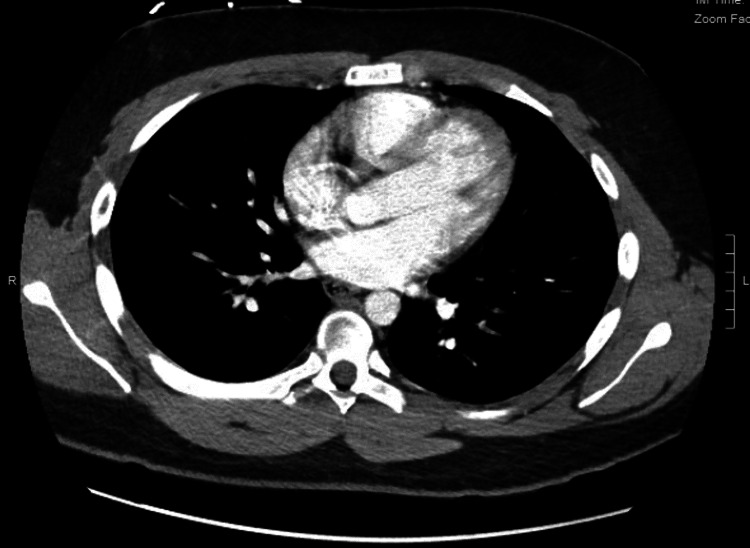
CT scan of the chest showing normal heart size. No pericardial effusion and unremarkable chest wall.

Chest CT scan showed normal heart size and no pericardial effusion (Figure 3).

**Figure 4:**
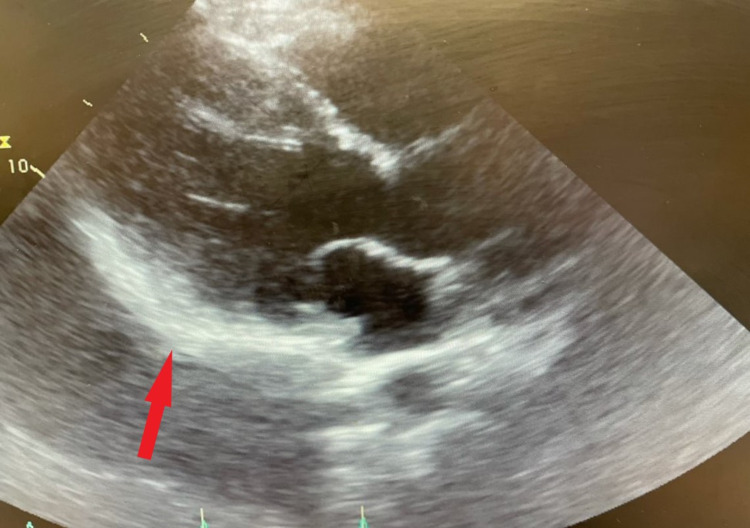
Echocardiogram showing epicardial brightness (red arrow) suggestive of pericarditis.

His echocardiogram showed epicardial brightness suggestive of pericarditis (Figure 4). No valvular abnormalities and normal ejection fraction estimated at 60-65%.

The patient was admitted and managed medically with colchicine 0.6 mg twice a day and aspirin 81 mg. Cardiology was consulted and a viral respiratory panel including Coxsackie A & B and adenovirus was performed, and they were negative. Repeat testing on day 2 showed decreased troponin I level to 2.18 ng/mL. The patient was discharged on day 4 after he improved clinically, and chest pain had been resolved. On discharge, he was given a prescription of colchicine 0.6 mg twice a day for 3 months and ibuprofen 600 mg three times a day for 1 week then taper it over the next 3 weeks. He was given instructions and appointments to follow up with a cardiologist and our family medicine residency clinic within 1 week of discharge.

## Discussion

We present a case of a young male adolescent with a clinical diagnosis of mild myocarditis without an infectious etiology following COVID-19 vaccination likely representing a rare adverse event to the vaccine. We encourage healthcare providers to be aware of this potential adverse event in young patients who present with cardiac symptoms after recent COVID-19 immunization. Back in April 2021, The Center for Disease Control and Prevention (CDC) issued a statement regarding the increased number of reported cases of pericarditis and myocarditis following the administration of COVID-19 vaccination. However, most of these cases were mild and responded well to medical therapy [5]. As of August 2021, there were 826 cases of pericarditis and myocarditis reported to the Vaccine Safety Adverse Event System (VAERS). Most of these cases were predominately in young male adolescents between the age of 18 and 24 [6]. In our literature review, we found several case reports and series that were published as of June 2021 of myocarditis following mRNA COVID-19 vaccine [3,7-9]. Most of these cases occurred predominately in young males who presented with chest pain 2 to 3 days following the administration of the second dose. All of them tested negative for the current SARS-CoV-2 and had elevated troponin and CRP. Most of them also showed ST elevation on ECG and had a few had abnormal echocardiogram with left ventricular ejection fraction (LVEF) <40%. The findings in those case reports were consistent with our patient’s presentation. Interestingly, our patient presented with chest pain 2 weeks after receiving the first dose of the mRNA COVID-19 vaccine. Almost in all case reports that were reviewed, patients had complete resolutions of symptoms at days 3-4 of their hospital course and were treated medically with anti-inflammatory and colchicine [7-9], similarly to what has been seen in our patient. Those findings suggest that the myocarditis-induced mRNA COVID-19 vaccine has a mild course and favors a good outcome. The predominance of the male gender in myopericarditis is still unclear [3]. It has been found in some studies that testosterone hormone may have a role in enhancing immunogenicity, thus leading to an increase in immune-mediated myocyte injury in myocarditis [3,10].

## Conclusions

We present a case of mRNA COVID-19-vaccine-induced myocarditis in a young male adolescent. Our case findings suggest a mild course of the disease with complete and fast recovery without sequelae. The reason behind male gender predominance in such cases is still unclear and given the significance of this topic during the COVID-19 pandemic, we recommend that further research should be directed to investigate the long-term effect of the mRNA COVID-19 vaccine-related myocarditis on cardiac function and further understand the immunopathogenic mechanism of the disease and its age and sex-related differences.
